# Serial Measurement of Serum Pancreatic Lipase Immunoreactivity, Feline Trypsin-like Immunoreactivity, and Cobalamin Concentrations in Kittens

**DOI:** 10.3390/vetsci9090469

**Published:** 2022-08-31

**Authors:** Evangelia M. Stavroulaki, Kassiopi Christina G. Kokkinaki, Manolis N. Saridomichelakis, Jörg M. Steiner, Jonathan A. Lidbury, Panagiotis G. Xenoulis

**Affiliations:** 1 Clinic of Medicine, Faculty of Veterinary Science, University of Thessaly, 224 Trikalon Str., 43132 Karditsa, Greece; 2 Gastrointestinal Laboratory, Department of Small Animal Clinical Sciences, Texas A&M University, College Station, TX 77843, USA

**Keywords:** PLI, TLI, B12, cat, gastrointestinal disease, pancreatitis, exocrine pancreatic insufficiency

## Abstract

**Simple Summary:**

Serum concentrations of feline pancreatic lipase immunoreactivity (fPLI), feline trypsin-like immunoreactivity (fTLI), and cobalamin are commonly used for the diagnostic investigation of cats with gastrointestinal signs. No information on these parameters in healthy cats less than 1 year of age exists. We aimed to evaluate serum concentrations of fPLI, fTLI, and cobalamin in healthy cats at different time-points during their first 12 months of life. Fourteen healthy 2-month-old kittens were included. Blood was collected at 2, 3, 4, 6, and 12 months of age, and serum concentrations of fPLI, fTLI, and cobalamin were measured. Serum fPLI and fTLI concentrations did not show any statistically or clinically significant differences in young kittens. In contrast, serum cobalamin concentrations were commonly below the reference interval in kittens. Serum fPLI and fTLI concentrations are not practically affected by age in kittens as young as 2 months of age and could be used for the investigation of pancreatic diseases.

**Abstract:**

Serum concentrations of feline pancreatic lipase immunoreactivity (fPLI), feline trypsin-like immunoreactivity (fTLI), and cobalamin are commonly used for the diagnostic investigation of cats with gastrointestinal signs. No information on these parameters in healthy cats less than 1 year of age exists. We aimed to evaluate serum concentrations of fPLI, fTLI, and cobalamin in healthy cats at different time-points during their first 12 months of life. Fourteen healthy 2-month-old kittens were included. Blood was collected at 2, 3, 4, 6, and 12 months of age, and serum concentrations of fPLI, fTLI, and cobalamin were measured. While there was a statistically significant difference in serum fPLI concentrations over time, there was no statistically significant difference between individual time-points. There was no significant difference in serum fTLI concentrations over time. Serum cobalamin concentrations were below the reference interval in 3/13 cats at 2 months of age and were significantly lower by 3 months, when 13/14 had hypocobalaminemia. By 12 months, serum cobalamin had significantly increased, yet 4/12 cats still had hypocobalaminemia. Serum fPLI and fTLI concentrations did not show any statistically or clinically significant differences in young kittens. In contrast, serum cobalamin concentrations were commonly below the reference interval in kittens. Serum fPLI and fTLI concentrations are not practically affected by age in kittens as young as 2 months of age and could be used for the investigation of pancreatic diseases.

## 1. Introduction

Serum concentrations of feline pancreatic lipase immunoreactivity (fPLI), feline trypsin-like immunoreactivity (fTLI), and cobalamin are commonly used for the diagnostic investigation of cats with gastrointestinal (GI) signs. Although GI and pancreatic diseases are more common in older cats, several studies have shown that certain conditions, such as food-responsive enteropathy (FRE), pancreatitis, and exocrine pancreatic insufficiency (EPI) can occur in young cats, even below the age of 1 year [1,2,3,4]. However, no information on serum fPLI, fTLI, and cobalamin concentrations is available in cats less than 1 year of age.

Measurement of serum fPLI concentration is considered to be the serum test of choice for the diagnosis of pancreatitis in cats, with a reported sensitivity ranging between 67% and 100% and a reported specificity of 82–100% [5,6,7,8]. Similarly, measurement of serum TLI concentrations is the diagnostic test of choice for EPI in both cats and dogs [9,10,11,12]. Serum cobalamin concentrations are commonly used for the evaluation of the absorptive capacity of the ileum, although its exact sensitivity and specificity are unknown [13,14,15]. Hypocobalaminemia is also commonly seen in cats with EPI, due to the absence of the intrinsic factor (IF) normally produced exclusively by the pancreas in this species [16]. In addition to its diagnostic significance, cobalamin has also therapeutic importance in cats, because its administration has been shown to improve the clinical signs and outcome in cats with chronic enteropathies [13].

Our hypothesis was that serum fPLI, fTLI, and cobalamin concentrations would not be affected by age in young cats. We aimed to evaluate serum concentrations of fPLI, fTLI, and cobalamin in cats at different time-points during the first 12 months of their life.

## 2. Materials and Methods

### 2.1. Ethics Approval

The study protocol was reviewed and approved by the Animal Ethics Committee of the University of Thessaly, Greece (AUP number: 54/13.2.2018).

### 2.2. Cats

A total of 23 domestic shorthair (DSH) kittens, approximately 2 months of age, were prospectively enrolled into the study, but only 14 of them were followed up until they reached 1 year of age. The age of the cats was estimated based on body size, dentition, and other physical characteristics [17,18,19].The enrollment of cats and the completion of the follow-up period took place between May 2018 and March 2020. All kittens were stray cats at enrollment and were either housed in foster homes or in individual cages at the Clinic of Medicine, at the Faculty of Veterinary Science of the University of Thessaly. All cats were eventually adopted into private homes before or by the end of the study and all owners signed an informed owner consent form.

### 2.3. Initial Evaluation of Cats and Inclusion Criteria

Upon admission, a thorough physical examination was performed in all cats, including evaluation of mental status, hydration status, body weight (BW), body condition score (BCS), gender, heart rate and rhythm, mucous membranes color, capillary refill time, respiratory rate, thoracic auscultation, abdominal palpation, and body temperature. In addition, all cats were kept at the Clinic to be observed for a few days, and the presence of diarrhea, vomiting, decreased or increased appetite, and other clinical signs were recorded. Cats were included in the study if they were deemed to be healthy, apart from mild conditions that are not expected to affect gastrointestinal function or serum fPLI, fTLI, and cobalamin concentrations (e.g., mild upper respiratory disease or mild dermatological problems). Cats were excluded from the study if they had concurrent health conditions that were severe enough to require specific treatments and/or hospitalization.

Prior to inclusion into the study, all kittens received the same antiparasitic treatment (fipronil, (S)-methoprene, eprinomectin, and praziquantel; Broadline, Boehringer Ingelheim International GmbH, Rhein, Germany) topically, and remained on the same monthly antiparasitic treatment throughout the study period. All were tested for feline leukemia virus (FeLV) antigen and feline immunodeficiency virus (FIV) antibodies (SNAP FIV/FeLV Combo test, IDEXX Laboratories, Westbrook, ME, USA) and for canine parvovirus antigen (SNAP Parvo test; IDEXX Laboratories, Westbrook, ME, USA), and were vaccinated (Purevax RCP and Purevax Rabies; Boehringer Ingelheim International GmbH) according to recent guidelines [20].

Finally, all cats consumed the same commercial diet (GEMON Cat Breeder Kitten; Monge & C. S.p.a., Torino, Italy) exclusively during the whole study period. The percentage of crude protein, crude oils and fats, crude fiber, crude ash, calcium, phosphorus, omega 6 essential fatty acids, and omega 3 essential fatty acids was 34%, 15%, 2.5%, 8.5%, 1.1%, 1%, 2.67%, and 1% respectively. The metabolizable energy was 4000 Kcal/kg dry matter.

### 2.4. Sample Collection and Follow-Up Period

For kittens ≤ 3 months of age, food was withheld for 6 h, while for kittens > 3 months of age food was withheld for 12 h prior to blood collection. Upon enrollment into the study, a total of 3.5 mL of blood was collected from each kitten from the jugular vein, allowed to clot for 30 min, centrifuged at 3000× *g* for 10 min, and serum was collected, placed into Eppendorf tubes, and stored at −80 °C until analysis. In addition to the initial blood sample collected at approximately 2 months of age, additional samples were collected at 3, 4, 6, and 12 months of age and were handled in the same manner as for the initial sample. Samples were stored for no more than 12 months before all parameters were analyzed. The samples were sent by overnight courier, packed with icepacks, and were analyzed at the Gastrointestinal Laboratory at Texas A&M University. All serum samples were thawed at room temperature and serum concentrations of each parameter (i.e., fPLI, fTLI, cobalamin) were measured on the same day.

On each sampling day, a detailed clinical history was obtained, and cats underwent a thorough physical examination, like on enrollment.

### 2.5. Assays

Feline PLI concentrations were measured by the Spec fPL assay (IDEXX Laboratories, Westbrook, ME) using a previously validated immunoassay [8]. The reference interval for fPLI is ≤3.5 µg/L, with 3.6 to 5.3 µg/L considered equivocal and >5.3 µg/L suggestive for pancreatitis [6,21,22]. Feline TLI concentrations were measured using a previously validated in-house radioimmunoassay (RIA) [12]. The reference interval for this assay is 12 to 82 μg/L, with concentrations ≤8.0 μg/L being diagnostic for EPI and 8–12 μg/L being equivocal [12,23,24]. The detection limit for fTLI in this assay was 1.9 μg/L. A validated automated chemiluminescence assay (Immulite 2000 Immunoassay System. Siemens Healthcare Systems GmbH, Erlangen, Germany) was used for cobalamin measurement [24]. The reference interval for cobalamin is 290 to 1500 ng/L.

### 2.6. Statistical Analysis

Data were tested for normality using the Kolmogorov–Smirnov test. Data were not normally distributed; therefore, overtime comparisons were performed with Friedman tests, while Dunn’s post hoc tests were performed to determine which time-points were significantly different. Statistical significance was set at *p* < 0.05. All statistical analyses were performed using statistical software packages (SPSS 23 for Windows, IBM Corp., Armonk, NY, USA; and Prism 9, GraphPad Software Inc., San Diego, CA, USA).

## 3. Results

### 3.1. Cats

A total of 14 cats completed the 10-month study. Of these, 8 were males and 6 were females. All cats were neutered/castrated by the end of the study period and were tested negative for FeLV antigen, FIV antibodies, and canine parvovirus antigen.

### 3.2. Serum fPLI Concentrations

**Figure 1:**
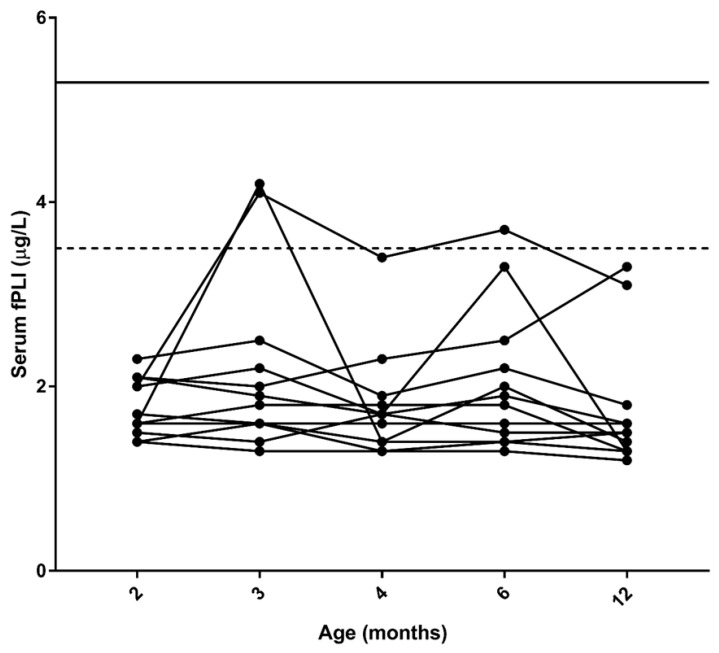
Serum feline pancreatic lipase immunoreactivity (fPLI) concentrations in the 12 cats included in the over-time analysis. The dashed line represents the upper limit of the reference interval (3.5 μg/L). The solid line represents the cut-off value for pancreatitis (5.3 μg/L).

Serum was available for all time-points for measurement of fPLI concentrations for 12/14 cats, and only these cats were included in the over-time analysis. Two additional cats were missing one time-point each (at 12 months). Overall, there was a statistically significant difference for serum fPLI concentrations over time (*p* = 0.0168; Figure 1). However, post hoc testing revealed that there was no statistically significant difference between any of the individual time-points. Overall, 0% (0/14) of cats had increased serum fPLI concentrations at 2 months of age; 21.4% (3/14, including 1 cat that was excluded from the over-time analysis) at 3 months; 0% (0/14) at 4 months; 7.1% (1/14) at 6 months; and 0% (0/12) at 12 months of age. All the increased serum fPLI concentrations were in the equivocal reference range (i.e., between 3.6 and 5.3 µg/L). Finally, no cats had consecutively increased serum fPLI concentrations over time (Figure 1).

### 3.3. Serum fTLI Concentrations

**Figure 2:**
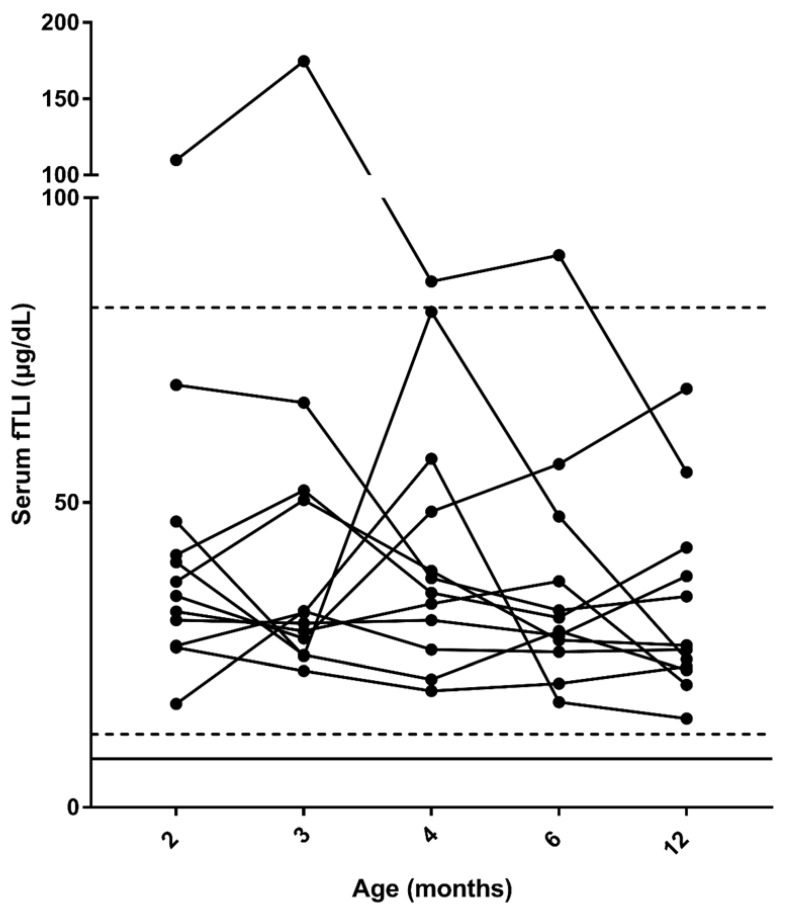
Serum feline trypsin-like immunoreactivity (fTLI) concentrations in the 12 cats included in the over-time analysis. The dashed lines represent the reference interval (12–82 μg/L). The solid line represents the cut-off value for exocrine pancreatic insufficiency (8 μg/L). Note that the *y*-axis is split.

Serum was available for all time-points for measurement of fTLI concentrations for 12/14 cats, and only these cats were included in the over-time analysis. One cat had two time-points missing (at 4 and 12 months) and another cat had one time-point missing (at 12 months). No significant differences of serum fTLI concentrations were found over time (Figure 2). In addition, no cats had serum fTLI concentrations below the lower limit of the reference interval at any time-point. In 21.4% (3/14, including the 2 cats that were excluded from the over-time analysis) of cats, fTLI was above the upper limit of the reference interval at 2 months; in 14.3% (2/14, including 1 cat that was excluded from the over-time analysis) at 3 months of age; while in 7.7% (1/13) and 7.1% (1/14) at 4 and 6 months of age, respectively. One cat had an increased fTLI concentration at both 2 and 3 months of age (this cat was not included in the over-time analysis) and one cat from 2 to 6 months of age (Figure 2).

### 3.4. Serum Cobalamin Concentrations

**Figure 3:**
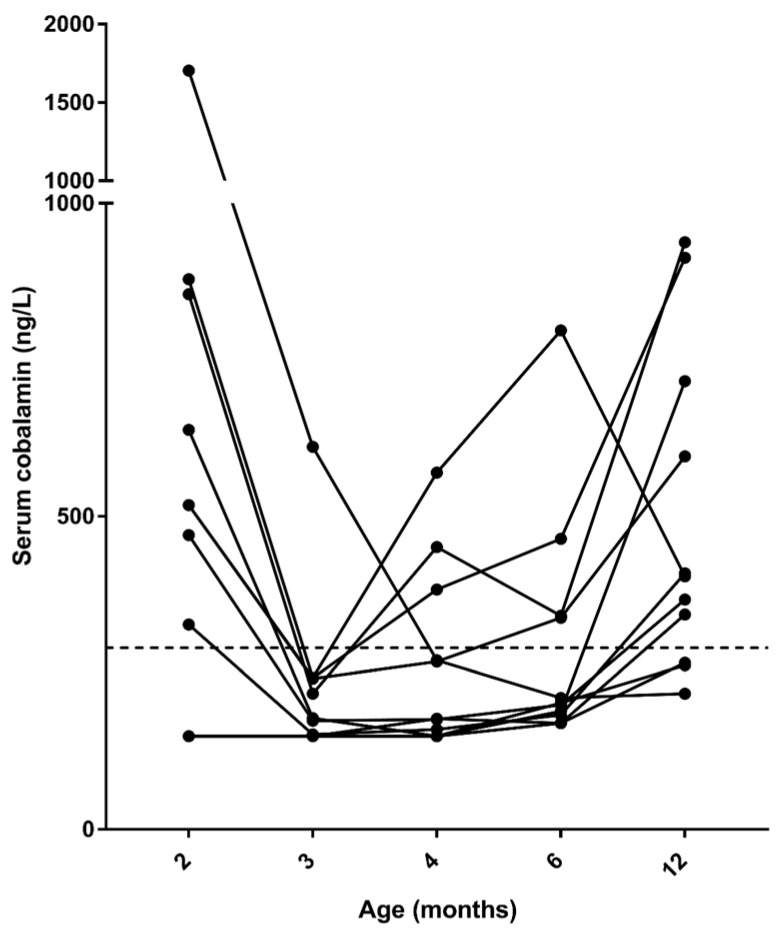
Serum cobalamin concentrations in the 11 cats included in the over-time analysis. The dashed line represents the lower limit of the reference interval (290 ng/L). Note that the *y*-axis is split.

Serum was available for all time-points for measurement of cobalamin concentrations for 11/14 cats, and only these cats were included in the over-time analysis. One cat had two time-points missing (at 4 and 12 months) and 2 cats had one time-point missing each (at 2 and at 12 months, respectively). Serum cobalamin concentrations significantly decreased (*p* = *0*.005) from 2 (median, 494 ng/L; range, <150–1704 ng/L) to 3 months of age (median, 178 ng/L; range, <150–611 ng/L), and this was followed by a significant increase (*p* < 0.001) by 12 months of age (median, 404 ng/L; range, 169–938 ng/L, Figure 3). Overall, 30.8% (4/13, including the three cats that were excluded from the over-time analysis) of cats had serum cobalamin concentrations below the lower limit of the reference interval at 2 months of age; 92.9% (13/14, including the 3 cats that were excluded from the over-time analysis) at 3 months of age; and by the age of 12 months, 33.3% (4/12, including one cat that was excluded from the over-time analysis) of cats still had cobalamin concentrations below the lower limit of the reference interval (Figure 3).

## 4. Discussion

Measurement of noninvasive markers of GI disease in cats with GI signs is routine in clinical practice. However, the concentrations of markers of GI and pancreatic disease have not been previously evaluated in clinically healthy cats less than 1 year of age. Thus, the aim of our study was to investigate the concentrations of three serum markers commonly used in the diagnostic investigation of GI diseases, namely serum fPLI, fTLI, and cobalamin concentrations, in young healthy cats. Overall, serum fPLI and fTLI concentrations were usually within the established reference intervals and were not affected by the age between 2 and 12 months. In contrast, serum cobalamin concentrations were significantly affected by age and different proportions of cats were hypocobalaminemic at different ages.

Serum fPLI concentrations were not associated with any clinically meaningful changes during the first year of life in our cats. In a small number of cats, serum fPLI concentrations were slightly above the upper limit of the reference interval at 3 and 6 months of age. However, the abnormally high serum fPLI concentrations in all these cats ranged between 3.6–5.3 μg/dL, concentrations that fall below the cutoff for a diagnosis of pancreatitis [21]. Serum fTLI concentrations did not change significantly during the first year of life in cats, and no cats had decreased serum fTLI concentrations, which are of interest for the diagnosis of EPI. A small percentage of cats had abnormally high serum fTLI concentrations, mainly at 2 and 3 months of age. From a total of 7 (of 67 measurements) increased serum fTLI concentrations, only one was associated with an abnormally high serum fPLI concentration (3 months of age, serum fPLI, 5 μg/L and fTLI, 144.3 μg/L). This cat was clinically healthy during physical examination, with no concerns reported by the owner, and no abnormalities of serum fPLI and fTLI concentrations were noted thereafter.

The activities and/or concentrations of pancreatic lipase and trypsin have not been thoroughly investigated in humans during early life, and different assays are used for evaluating these parameters [25]. The exocrine pancreas in humans has been described to mature slowly following birth, which is associated with low activities of lipase and trypsin in the pancreatic juice as well as with reduced response to secretagogues in 1-month-old infants [26]. In one study, the activities of lipase and trypsin measured in duodenal aspirates increased significantly in children from 1.7 to 8 years of age, suggesting an ongoing pancreatic secretory maturation process [27]. In our study, fPLI and fTLI concentrations did not significantly change with age, and the cats with concentrations higher than the upper limit of the reference interval did not have clinical signs of pancreatitis. Therefore, these values may represent the individualized maturation process of the secretory exocrine pancreas. Alternatively, since serum PLI concentrations show important intraindividual variability in dogs, this may also be the case in cats and may have been a contributing factor for the increased concentrations noticed in our study [28].

Serum cobalamin concentrations were subnormal in a considerable percentage of cats at all time points (ranging between 30% and 93%). In addition, serum cobalamin concentrations significantly decreased between 2 and 3 months of age, and this was followed by a gradual and significant increase by the age of 1 year. In the only study published so far as a full paper where the relationship between age and serum cobalamin concentrations was investigated, only adult (between 3 and 9 years of age) healthy colony cats were included [29]. In that study, an inverse relationship between serum cobalamin and age was identified, with increasing age being significantly associated with decreasing serum cobalamin concentrations.

In humans, serum cobalamin concentrations undergo significant changes during development. During early life, serum cobalamin decreases in humans, with the lowest concentrations observed from 6 weeks to 6 months of age, and this is followed by a gradual increase with a peak between 3 and 7 years of age, while a reduction is observed thereafter, which reaches a plateau towards adulthood [30,31]. Several studies have been published in humans suggesting the necessity to establish different reference intervals for serum cobalamin concentrations in infants and children, instead of the ones used in adults [30,32,33,34,35], because low serum cobalamin concentrations (based on adult reference intervals) can be a common and normal finding in breast-fed infants [33,36]. Similarly, based on the present study, the use of the cobalamin reference interval of adult cats may not be appropriate in kittens and young cats. This should be taken into consideration when deciding whether hypocobalaminemia in young cats reflects a clinically relevant deficiency that requires cobalamin supplementation or whether it is a physiological process.

The mechanisms of cobalamin absorption, transport, and metabolism have not been elucidated in cats during early life. In humans, cobalamin is stored in the liver of the fetus during pregnancy, and subsequently, hepatic cobalamin stores play a major role in determining serum cobalamin concentrations during the first months of newborn life [36,37]. Following birth, cobalamin is supplemented through the milk along with cobalamin binders, especially haptocorrin or R-protein, that prevent cobalamin uptake by gastrointestinal bacteria [38].

During infancy in humans, the IF is either not produced in sufficient quantities by the stomach or it is not functional until a certain age [39,40,41]. In addition, IF receptors in the ileum are not adequately expressed due to the immaturity of the GI tract [42]. Finally, gastrin and pepsin, which decrease the gastric pH allowing the release of cobalamin from ingested proteins, are also not produced in sufficient amounts [42]. Therefore, cobalamin transport in infants is reported to be mediated by haptocorrin, and the absorption of cobalamin is suspected to take place by alternative receptors until the IF and IF-receptors are adequately developed [42]. In cats, IF is almost exclusively produced by the ductal cells of the exocrine pancreas [15] and it is unknown when the pancreatic IF production and ileal IF-receptor expression start playing major roles for cobalamin transport and absorption following birth. All cats remained clinically healthy over the 10-month follow-up period in our study, and no concerns were reported by their owners. Therefore, the reduction in cobalamin between 2 and 3 months of age as well as the high prevalence of hypocobalaminemia until the age of 6 months may reflect an age-related process driven by the immaturity of the GI tract and/or reduced pancreatic IF secretion, and the transition from breastfeeding/milk-replacement formula to a solid diet.

Serum cobalamin concentrations are also known to be affected by the intestinal microbiota [43]. The intestinal microbiota and metabolome have recently been shown to change significantly from 2 to 12 months of age [44,45]. The immaturity of the intestinal microbiome in young kittens until they reach an adult-like state might be an additional contributing factor that could be related to the changes in serum cobalamin concentrations during the first year of life in cats.

Markers of cellular deficiency of cobalamin, such as serum methylmalonic acid (MMA) concentrations, have been previously investigated in cats [46,47,48]. Measurements of serum MMA concentrations could have been useful in our study, although they have not been evaluated in young kittens and it is unknown if they represent an accurate marker of cellular cobalamin deficiency. However, MMA is measured by gas chromatography–mass spectrometry (GC-MS) and requires a large volume of serum that was not available in our study.

The main source of cobalamin in cats is considered to be the diet [29]. All cats in our study were fed the same diet, and therefore, differences in dietary cobalamin can be excluded as a possible cause of the differences in cobalamin concentrations among cats. In addition, the fact that serum cobalamin concentrations fluctuated over time without any diet changes suggests that dietary factors are unlikely to be responsible for these changes.

Food was withheld for 6 h in kittens < 3 months of age and for 12 h for kittens > 12 months of age. This was in order to prevent problems such as hypoglycemia, caused by prolonged fasting in kittens of a very young age. However, this is not expected to have influenced our results, because in a recent study it was shown that feeding did not affect serum fPLI and cobalamin concentrations [49]. Although feeding can affect serum fTLI concentrations, no differences were found in our study for fTLI over time.

Our study had some limitations. The number of cats that completed the study was relatively small. This was a prospective study with a relatively long follow-up period, and it was challenging to engage owners to regularly come back for blood collection. Even with these small numbers, our results were rather consistent in each group, and therefore, they likely reflect the general population.

## 5. Conclusions

In conclusion, our results indicate that serum fPLI and fTLI concentrations are not significantly affected by age in young kittens, between the ages of 2 and 12 months, and can be used for the investigation of pancreatic diseases in these animals. In contrast, serum cobalamin concentrations are commonly below the established reference interval in kittens. Whether this represents a physiological or a pathological condition is not clear, and further studies are needed to investigate this finding. In any case, an age-specific reference interval for serum cobalamin concentration should be established for kittens. However, given that cobalamin supplementation is not associated with any known side effects, overdiagnosis of hypocobalaminemia in kittens would not be expected to be detrimental.
